# Comparative Morphology of Skeletal Development in *Homo sapiens* and *Raja asterias*: Divergent Stiffening Patterns Due to Different Matrix Calcification Processes

**DOI:** 10.3390/ani14172575

**Published:** 2024-09-04

**Authors:** Ugo E. Pazzaglia, Piero A. Zecca, Genciana Terova, Fabrizio Serena, Cecilia Mancusi, Giovanni Raimondi, Guido Zarattini, Mario Raspanti, Marcella Reguzzoni

**Affiliations:** 1 Department of Medical and Surgical Specialties, Radiological Sciences and Public Health, University of Brescia, 25123 Brescia, Italy; guido.zarattini@unibs.it; 2 Department of Medicine and Technological Innovation, University of Insubria, 21100 Varese, Italy; pieroantonio.zecca@uninsubria.it (P.A.Z.); marcella.reguzzoni@uninsubria.it (M.R.); 3 Department of Biotechnology and Life Sciences, University of Insubria, 21100 Varese, Italy; 4 Institute of Marine Biological Resources and Biotechnology, National Research Council (CNR-IRBIN), 80131 Mazara del Vallo, Italy; fabrizio50serena@gmail.com; 5 Environmental Protection Agency of Tuscany Region (ARPAT), 56127 Pisa, Italy; c.mancusi@arpat.toscana.it; 6 Aquarium of Livorno, 57127 Livorno, Italy; graimondi@costaedutainment.com

**Keywords:** cartilage anlagen development, endochondral ossification, Batoidea calcifying cartilage, “crustal” and “catenated” tiles pattern

## Abstract

**Simple Summary:**

Before calcification, the early skeletal development of both *Homo sapiens* and the chondrichthyan fish *Raja asterias* is exclusively cartilaginous. This cartilage is formed through tissue segmentation and chondrocyte mitoses. Mineral deposition in the cartilage matrix influences the stiffness and shape of the skeletal segments. In mammals, calcified cartilage serves as a scaffold for bone deposition, which is then remodeled. Conversely, chondrichthyans retain calcified cartilage as their skeletal structure, forming calcification nuclei or “tesserae”. These structures adapt to aquatic locomotion. In mammals, endochondral ossification provides limb bones with the necessary stiffness for terrestrial locomotion. X-rays of marine mammals show how endochondral ossification in dolphin flippers adapts to aquatic demands, including shortening of certain bones and an increase in elements in the autopodium’s central rays.

**Abstract:**

Before calcification begins, the early embryonic and fetal skeletal development of both mammalian *Homo sapiens* and the chondrichthyan fish *Raja asterias* consists exclusively of cartilage. This cartilage is formed and shaped through processes involving tissue segmentation and the frequency, distribution, and orientation of chondrocyte mitoses. In the subsequent developmental phase, mineral deposition in the cartilage matrix conditions the development further. The stiffness and structural layout of the mineralized cartilage have a significant impact on the shape of the anlagen (early formative structure of a tissue, a scaffold on which the new bone is formed) and the mechanical properties of the skeletal segments. The fundamental difference between the two studied species lies in how calcified cartilage serves as a scaffold for osteoblasts to deposit bone matrix, which is then remodeled. In contrast, chondrichthyans retain the calcified cartilage as the definitive skeletal structure. This study documents the distinct mineral deposition pattern in the cartilage of the chondrichthyan *R. asterias*, in which calcification progresses with the formation of focal calcification nuclei or “tesserae”. These are arranged on the flat surface of the endo-skeleton (crustal pattern) or aligned in columns (catenated pattern) in the radials of the appendicular skeleton. This anatomical structure is well adapted to meet the mechanical requirements of locomotion in the water column. Conversely, in terrestrial mammals, endochondral ossification (associated with the remodeling of the calcified matrix) provides limb bones with the necessary stiffness to withstand the strong bending and twisting stresses of terrestrial locomotion. In this study, radiographs of marine mammals (reproduced from previously published studies) document how the endochondral ossification in dolphin flippers adapts to the mechanical demands of aquatic locomotion. This adaptation includes the reduction in the length of the stylopodium and zeugopodium and an increase in the number of elements in the autopodium’s central rays.

## 1. Introduction

Mineral deposition in the organic matrices of both Mammalians and Chondrichthyes plays a crucial role in skeletal growth, development and function. While calcification can occur in any matrix of living tissues due to degeneration or necrosis [1], cartilage and bone are unique in that their mineral deposition processes are precisely controlled and coordinated with the growth progression and function of the skeletal segments. This control provides the necessary stiffness for protection from the external environment and to meet the mechanical demands of locomotion. Although bone and matrix calcification patterns differ, in both mammals and cartilaginous fishes, skeletal segments differentiate within the median embryonic mesoderm layer. Here, cartilage anlagen are formed, with their shape being influenced by the distribution, frequency, and orientation of chondrocyte mitoses before mineral deposition begins [2,3,4]. In the subsequent fetal developmental phase, mineral deposition in the tissue matrix also plays a crucial role in the growth of the cartilage anlage and the final shape of skeletal segments.

Recognizing the homology between fish fins and tetrapod limbs [5], this study compares the early skeletal development of *Homo sapiens* with that of the chondrichthyan *Raja asterias*. The choice to morphologically compare two species so distant and evolutionary divergent is motivated by their different limb and fin stiffening patterns, which are adapted to movement on land and in the water column, respectively [6].

Additionally, supplementary data from dolphin flipper radiographs, reproduced from a previously published study [7], have been used to examine the effects of the marine environment on the pattern of endochondral ossification. Mammalian embryonic and fetal development is the most extensively studied model compared to all other vertebrates. In contrast, Chondrichthyes, with a much smaller number of species than Osteichthyes, offer a unique opportunity for a comparative histo-morphological analysis of skeletal stiffening patterns, shedding light on the different mechanical requirements of locomotion on land and in the water column.

In a 5-week-old human embryo approximately 1 cm in length, corresponding to stage 16 [8], the buds of the upper and lower limbs can be observed. Over the next 3–4 weeks, the anatomical positions of the corresponding skeletal segments become identifiable [9]. For Chondrichthyes development, Maxwell et al. [10] provided a reference table for stage classification of the skate *Leucoraya ocellata*, which was used as a rough reference for the histo-morphological comparison between *H. sapiens* and the Batoidea *R. asterias*.

Both species exhibit similar modeling of cartilage anlagen before calcification and early mineral deposition in the cartilage matrix; however, they diverge in the organization of the calcified skeletal framework. Considering the role of natural selection in species evolution, comparing the un-remodeled calcified cartilage with the more prevalent endochondral ossification (in terms of species diversity) can enhance our understanding of the relationship between mechanobiology and the structural evolutionary dynamics of the calcifying skeleton. Relevant data on marine mammals’ skeletal development from the scientific literature were also considered pertinent to this study [11,12,13,14,15].

## 2. Materials and Methods

### 2.1. Ethics Statement

The study protocol received approval from the Council of the Department of Medical and Surgical Specialties, Radiological Sciences, and Public Health at the University of Brescia (approval code: 171.3; approval date: 7 May 2015).

### 2.2. Source of the Material

The material for this study was sourced from the following:

1. ***Homo sapiens* Embryonic and Fetal Stages**: Data were derived from Streeter’s illustration [9] based on autopsy material from the Carnegie Institution of Washington.
2. ***Leucoraya ocellata* Embryonic and Fetal Stages**: Data were taken from the illustrations by Maxwell et al. [10].
3. **Histological slides of Human Fetal Limb Skeletal Segments** were taken from the Archive of Morbid Anatomy (Spedali Civili di Brescia), where all pregnancy terminations between the 16th and 22nd week of gestation were routinely examined and archived (corresponding to Streeter stages 22–23). The selection of slides used in the present study was based on initial prenatal ultrasound diagnosis to exclude skeletal malformations. Two previous studies using the same histological material have been published [3,4] with the study protocol approved by DSMC Ethic Committee of the Brescia University (171.3/7 May 2015).
4. ***Raja asterias* juvenile specimens**: Twelve juvenile specimens (Table 1) were provided by Aquarium of Livorno (Italy). These specimens were born from internally fertilized eggs, which were anchored in shallow corners. After oviposition from the capsule (from September 2022), the juveniles were reared in seawater in an indoor fishpond at a temperature of 18 °C. Embryonic development (not documented in this study) lasted 2–6 months [16]. The approximate age of the examined juveniles was 2 months (specimen 1); 3–5 months (specimens 2–8); and over 6 months (specimens 9–12). Overnight dead specimens were collected in the morning and immediately fixed in a 10% buffered formaldehyde solution. Older *R. asterias* specimens (specimens 13–15) were collected during a scientific campaign conducted by ARPAT team (U.O. Risorsa Ittica e Biodiversità Marina, Livorno) in the waters of Tuscany (Northwestern Mediterranean Sea) and fixed in formaldehyde solution within 24 h from capture.
5. **Data on marine mammals’ pectoral fin (flipper) skeletal development** were derived from published radiographic film images of bottlenose dolphin (*Tursiops truncatus*) pectoral flipper bone maturation stages from [7] for comparison with the ossification pattern of human upper limb skeletal system.

### 2.3. Selection and Processing of Human Specimens

The inclusion criteria for histological slides of upper limb skeletal segments of *H. sapiens* (from 16 to 22 weeks gestation abortions) in the study were that the sections showed complete cartilage anlage or, in the case of a partial segment, the longitudinal or transverse plane could be assigned. A total of 59 histological slides from 12 fetuses met these criteria [4]. All showed the same pattern of chondrocyte maturation, corresponding to histological stages 3b-7 according to the classification of Rivas and Shapiro [17].

### 2.4. Processing of R. asterias Specimens

The pectoral fins were separated from the axial endoskeleton by cutting the pectoral girdle on the midline. Dorsal/ventral skin and muscles were removed, and radiographs were taken in dorso-ventral projection to document the calcified skeletal architecture of the fin. Only a skin band of about 1.5 cm in length was left to avoid damaging the apical most radials and the fine subcutaneous keratin fibers. Unstained specimens of the medial and lateral fin sectors were transilluminated in Petri dishes (in phosphate buffer) at low magnification using an Olympus SZX7 Olympus stereomicroscope (Olympus Ltd., Milan, Italy), or heat-deproteinated at 400 °C and mounted on glass slides. Heat-deproteination is a technique for viewing calcified tissue: the slide is held in a muffle at 400 °C for 24 h. The heat burns off all organic components and highlights the mineralized structures. Other fin samples were decalcified for 30 days in 2% acetic acid-hydrochloric acid, embedded in paraffin, stained as previously mentioned, and mounted on glass slides for observation with an Olympus BX51 microscope (Olympus Ltd., Milan, Italy). Non-decalcified samples were embedded in Technovit 7200 resin (Kulzer GmbH, Leipzig, Germany), sectioned to a thickness of approximately 150 µm using the Exact cutting-grinding system (Exact Advanced Technology GmbH, Norderstedt, Germany) and stained with von Kossa or methylene blue acid fuchsin.

### 2.5. Raja asterias Pectoral Fin Radiograph Morphometry 

Digitized radiographic film images in dorso-ventral projection of the right pectoral fin of older specimens were taken after dissection of the skin and muscle layers. Nine central radial lines from the pterygial arch to the lateral margin were measured using the software program Cells (Soft Imaging System GmbH, Munster, Germany). The analysis focused on the mean length of the radials between the medial and lateral sectors, using the transverse line of radial duplication as a reference, and on the variation in length along the medial–lateral sequence. The radials of the medial sector were numbered from 8 to 1, starting with the radials characterized by the rotation of the columns (reference plane 9 in the diagram). The single-column radials of the lateral sector were doubled, and the measurements were made by the homologous radial length sum/2, numbered from 10 to 16 in the direction of the outer edge, while the length of the most apical radials was not measurable in the radiographs.

## 3. Results

### 3.1. Early Cartilage Anlagen Development

Early cartilage anlagen of skeletal segments become distinguishable after chondrocytes differentiation in the embryonic mesenchyme of limbs buds in *H. sapiens* (from Streeter stage 16, age about 6 weeks) and in the Batoidea. The initial shape of the skeletal cartilage anlagen is undefined, but by Streeter stage 20 in *H. sapiens* embryos, their number and position are established [9]. Maxwell et al. [10] documented the differentiation of cartilage anlagen in the pectoral fins’ radials of *Leucoraja erinacea* (Batoidea), supporting early homology between tetrapod’s limb and fin development [5,18]. The number and shape of the embryonic cartilage anlagen are determined by species-specific gene networks and associated cascades influencing chondrocyte mitoses frequency and spindle orientation [19,20].

**Figure 1:**
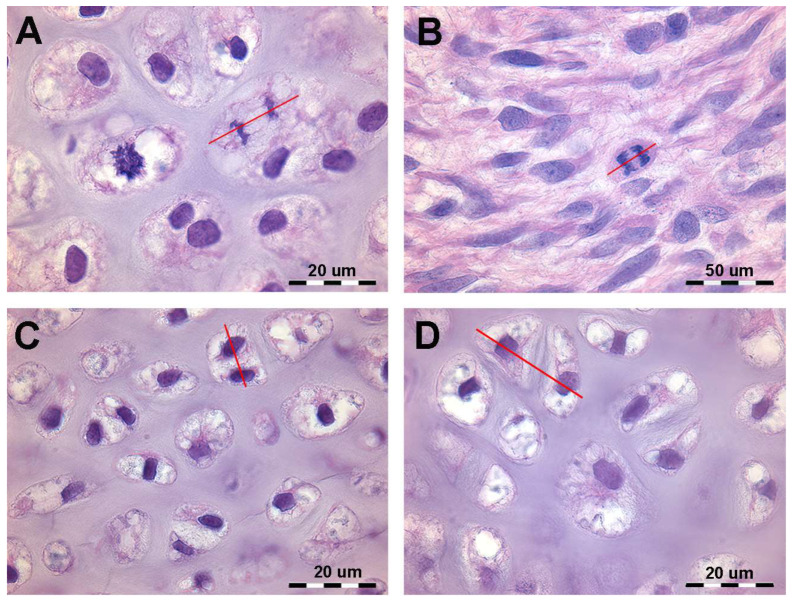
Histology of the autopodial cartilage anlagen growth of *Homo sapiens* (hematoxylin–eosin, dec. sections). (**A**) Mitotic chondrocyte phase with dissolution of the nuclear membrane, doubling of the chromosomes and attachment to the spindle (prophase and metaphase). The red line shows the axis of the mitotic spindle. (**B**) Migration of the doubled chromosomes towards the spindle poles (anaphase). The red line shows the axis of the mitotic spindle. (**C**) Duplicated chondrocytes still in a single lacuna. The red line shows the axis of the duplicated chondrocytes. (**D**) Paired chondrocytes have formed their own lacunae, separated by matrix septa of increasing thickness. The red line shows the axis of the recently duplicated chondrocytes that have formed their own lacunae.

During mitosis, chromosomes attach to the spindle’s equatorial plane (prophase), nuclear membrane dissolve, and spindles elongate (metaphase), guiding chromosomes to spindle poles (anaphase). Chromosomes then compact at spindle ends, a new nuclear membrane forms, and the spindle disappear (telophase). Cytoplasmic separation completes daughter cell formation with identical chromosomes and genes (Figure 1). Histomorphology of developing cartilage anlagen supports this modeling pattern based on the (a) orientation of the duplicating chondrocytes spindle in the cartilage tissue volume of the bud and (b) mitotic frequency and zonal distribution characterizing growth and shaping limb cartilage anlagen in vertebrates. A sufficiently comprehensive analysis of mitotic alignment is difficult to evaluate in histological specimens because mitotic chondrocytes can only be documented in the metaphase when the spindle lays in the plane of the section, requiring thin sections (about 7 µm, high magnification) (Figure 1B). The sensitivity of the method can be increased by considering advanced stages of chondrocyte duplication, such as the orientation of chondrocyte pairs in a single or paired lacunae separated by a flat cartilage matrix septum (Figure 1C,D).

### 3.2. Human Fetal Upper Limb Histology

**Figure 2:**
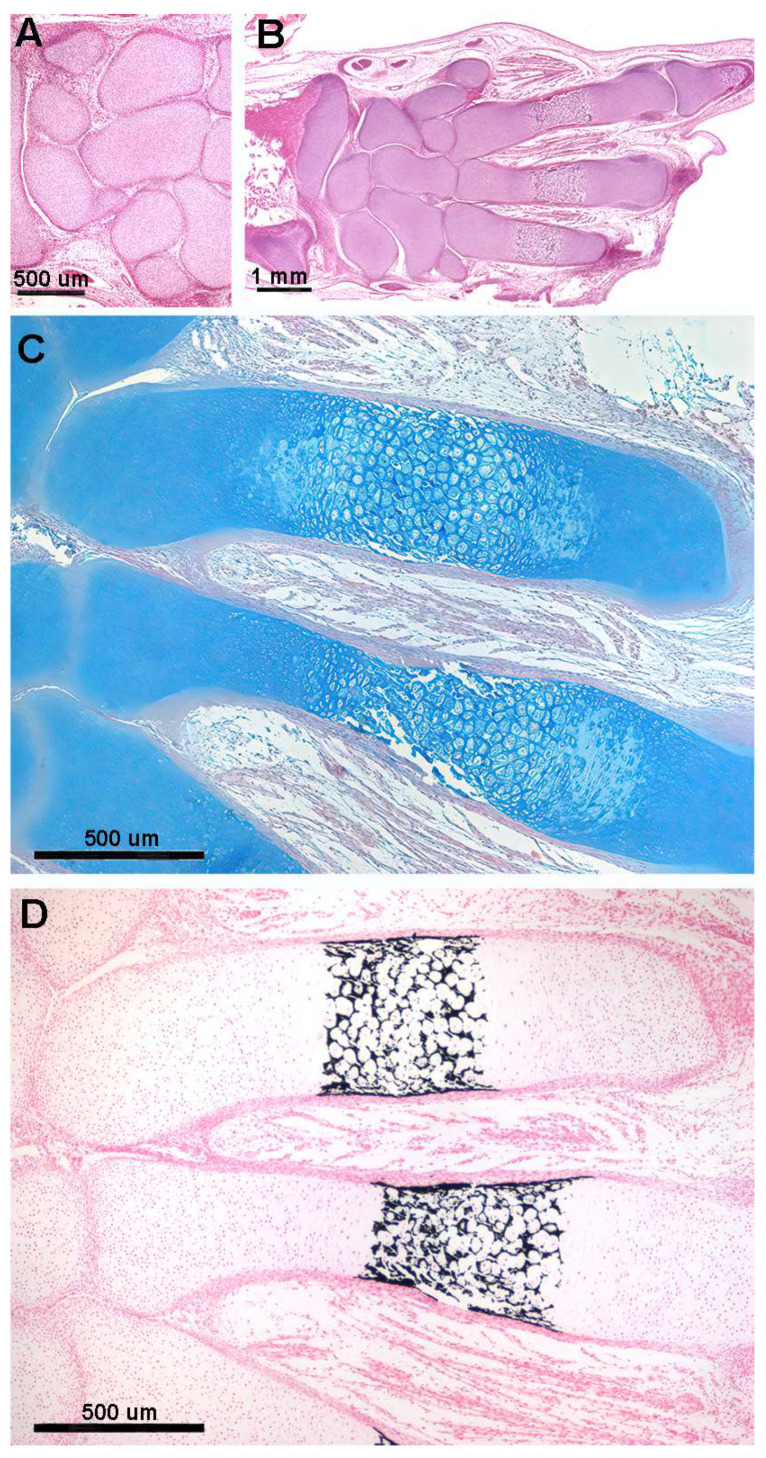
Histology of the fetal autopodium of *Homo sapiens*. (**A**,**B**) (hematoxylin–eosin, dec. section) Form of the autopodium elements, with incipient mineral deposition in the middle sector of the metacarpals, not yet initiated in the short carpal bone anlagen and in the epiphyses. (**C**) (Alcian-blue, dec. section) Initial phase of mineral deposition with swelling of the chondrocytes (usually referred to as hypertrophy). At this stage, there are no signs of epiphyseal calcifying centers forming. (**D**) (Von Kossa-neutral red, un-dec. section) Mineral deposits in the cartilage matrix between the swollen chondrocytes and early mineral deposits in the periosteal bone envelope.

Histology of human fetal upper limb elements (Streeter stages 22–23) before calcification shows distinct globular cartilaginous elements of the carpus and cylindrical phalanges of the fingers (Figure 2A,B). The long bones (humerus, radius, ulna and metacarpals) form from a median cylindrical segment (diaphysis) combined with globular ossification centers at the extremities (epiphyses) (Figure 2C,D). This supports a modeling pattern with a (i) regular and balanced 3D distribution of mitotic spindles orientation in epiphyses and (ii) preferential zonal distribution of longitudinally oriented mitoses in diaphysis.

Mineral deposits in the matrix scaffold (stiffening of the fetal appendicular skeletal elements) can initially occur in the middle of the long segment anlagen in Chondrichthyes, tetrapods and birds as well as in *H. sapiens* fetal limbs (homologous to the pectoral and pelvic fins of Rajidae), with initial mineral deposition occurring in the middle of the cartilaginous anlagen of the long bones and progressing proximally and distally, while the epiphyseal cartilage remains completely uncalcified until the later appearance of the independent epiphyseal ossification centers. These demarcate the metaphyseal growth plate cartilages (the anatomical structures of the long bones that control longitudinal growth in the juvenile phase).

**Figure 3:**
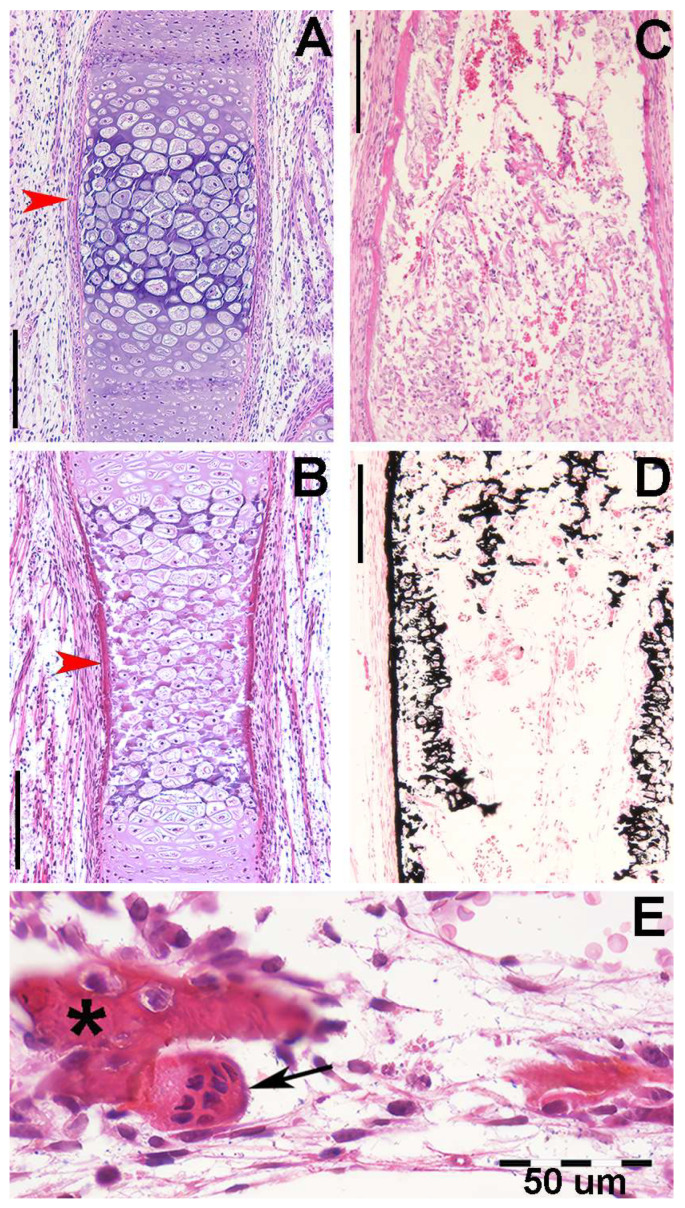
Histology and primary calcified cartilage matrix resorption in *Homo sapiens* autopodium metacarpals. (**A**) (hematoxylin–eosin, dec. section) Swollen chondrocytes of the central anlage zone with matrix more densely stained by hematoxylin where mineral deposition occurs. Initial formation of the bone periosteal sleeve (red arrow). (**B**) (hematoxylin–eosin, dec. section) Vascular invasion and reabsorption of calcified cartilage matrix in the central zone of the anlage; the upper and lower sectors retain the denser colored matrix. Increased thickness of the bone periosteal sleeve (red arrow). (**C**) (hematoxylin–eosin, dec. section, bar = 100 μm) Advanced vascular invasion and calcified matrix resorption in the central zone of the anlage. (**D**) (Von Kossa neutral red, un-dec. section, bar = 100 μm) Residual calcified cartilage matrix of the central zone and first cortical bone layer of the diaphysis. (**E**) (hematoxylin–eosin, dec. section) Bone matrix (*) of the trabecula, resorbing two osteocytes, and a multinuclear osteoclast (black arrow) remodeling a metaphyseal trabecula.

The cartilage anlagen of the carpal and tarsal elements undergo the same process of mineral deposition in the cartilage matrix, but their shape is determined by the random orientation of the duplicating chondrocytes. The subsequent stages of mineral deposition in tetrapods, birds, and mammals take place in the already calcified cartilage scaffold between hypertrophic chondrocytes or on the outer surface of the fetal skeletal segment (Figure 3A,B). However, this later mineral deposition must be distinguished from the embryonic one because (i) the protein matrix in which the mineral crystals are deposited is different (collagen type 2 and other protein molecules synthetized by osteoblasts); (ii) the cells controlling mineral deposition are osteoblasts and not chondrocytes; and (iii) other biological processes are involved, such as resorption of the calcified cartilage matrix by osteoclasts, vascular invasion and differentiation of osteoblasts from the mesenchyme (reflected by the terms “remodeling” and “endochondral ossification”). These processes are maintained throughout the life cycle of individuals of the species (Figure 3C–E).

### 3.3. Raja asterias Fin Development and Columnar Patterns

**Figure 4:**
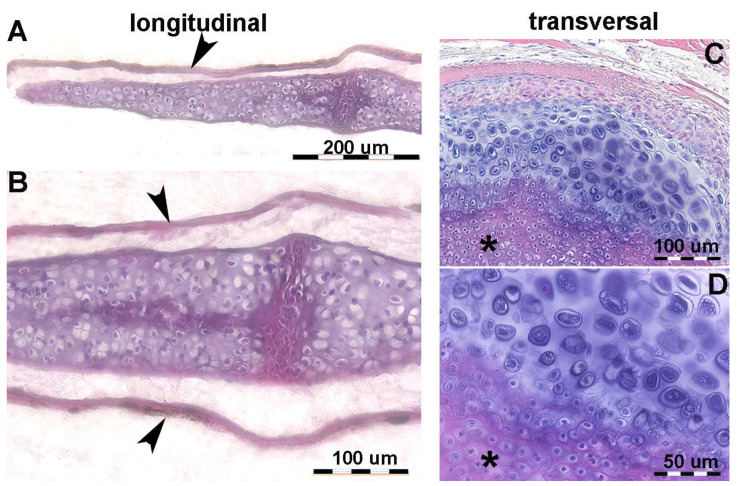
Histology of *R. asterias* pectoral fin radials. (**A**,**B**) (hematoxylin–eosin, dec. longitudinal section) High density of duplicating chondrocytes within a single lacuna and paired lacunae developing the radial (cylindrical) cartilage anlage. The matrix stained more intensely with hematoxylin corresponds to the zones of impendent mineral deposition that occur along the central axis of the radialis or in correspondence with the inter-radial amphiarthroses. Black arrows indicate the tegument. (**C**,**D**) (hematoxylin–eosin, dec., transverse section) Swellings of chondrocytes with densely stained matrix reproduce the histological features of mineral deposition of *H. sapiens* anlagen and show the non-remodeled texture of the calcified cartilaginous skeleton of chondrocytes (left section of figures). (*) the central cartilage core of the radials.

In *R. asterias*, the radial cylindrical shapes are determined by the orientation, number, and distribution of chondrocyte mitosis in the 3D model volume prior to calcification (Figure 4A,B), similar to human limb anlagen, but with some differences in terms of mineral deposition. Initial calcification occurs at the ends of the radial cartilage cylinders, forming medial and lateral plates with focal mineral deposits along the central axis forming aligned columnar plates. Transverse sections through the radial anlage (Figure 4 C,D) show a peripheral non-calcified cartilage muff and an internal calcified column that varies between round or oval shapes in the medial or lateral fin sectors.

**Figure 5:**
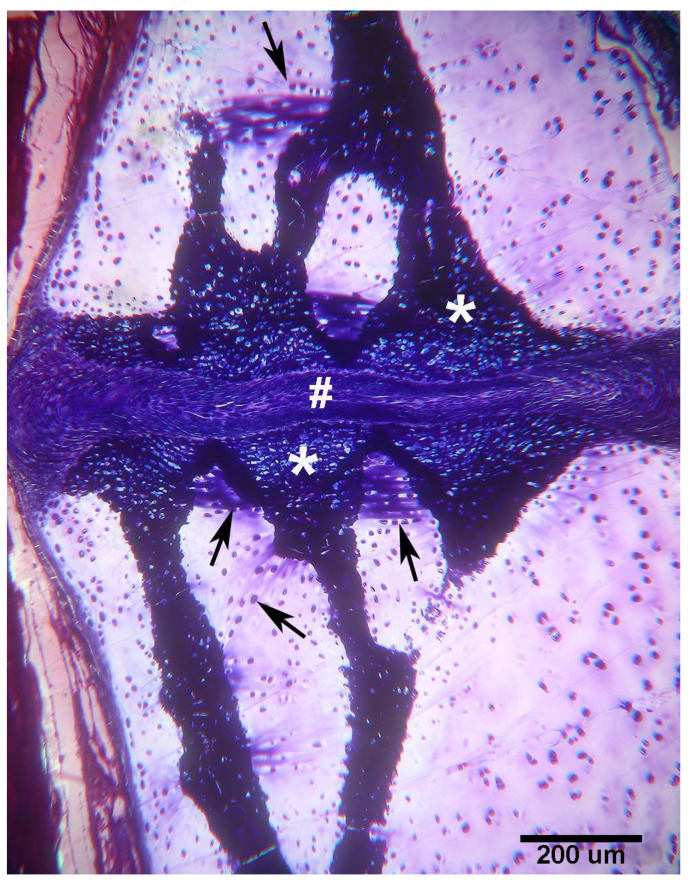
*Raja asterias* mature specimen of the inter-radial joint (toluidine blue, not declined/embedded in resin, longitudinal section). The joint consists of a layer of connective tissue (#) lying between the two discs (*) of calcified cartilage held by the branches of the central columns of the radialis. In the non-calcified cartilage matrix (weakly stained with toluidine), the tendency of the chondrocytes to align themselves into columns can be seen (arrows).

The stiffness of the radial segments varies with the number of inner columns (mono-bi- or multi-columnar). Histological analysis shows that early mineral deposition occurs in the matrix around voluminous chondrocytes, but with a different morphology from that of the “hypertrophic cells” observed in the metaphyseal growth plate cartilage of the long bones of *H. sapiens* [21]. In all developing radial units, a common structural arrangement can be observed in which the area of the uncalcified cylinder corresponds to that of the upper and lower disc (Figure 5). The cross-sectional area of the transversely cut calcified columns is smaller than that of the entire radial, while the disc (perpendicular to the radial axis) is stabilized by columnar bifurcations at the ends (Figure 5). 

**Figure 6:**
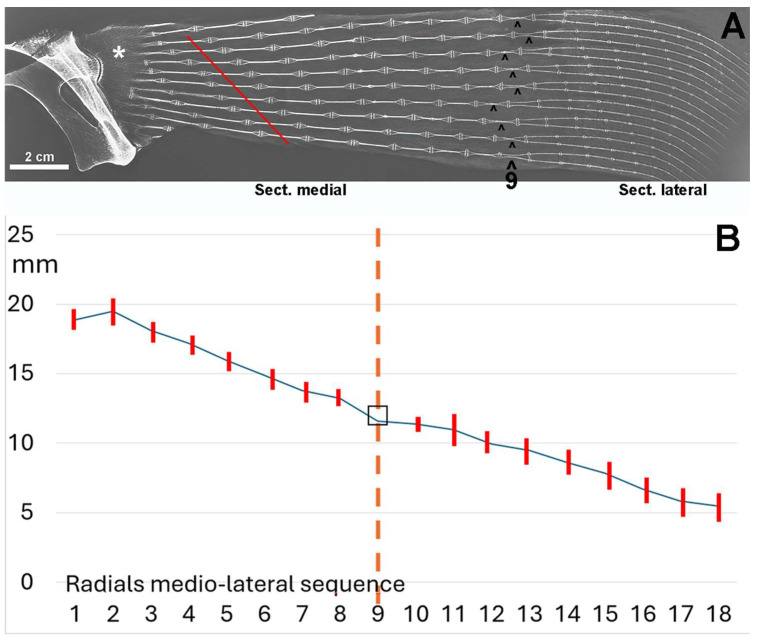
Radiograph/morphometry of the central sector of the pectoral fin in dorso-ventral projection of an adult specimen of *R. asterias*. (**A**) The medial and lateral sectors are distinguished by the radials, whose two columns (superimposed in the dorso-ventral plane) run horizontally approximately halfway along the length of the fin. This diagram, reproduced from [6], illustrates the irregular basal row of radial joints in the pterygium, highlighting some columns that are fused to the pterygium (*). The diagonally scaled rows of homologous radials (red line) reflect the curved profile of the entire fin margin and the gradual reduction in the number of radials in the anterior and posterior part of the fin. The symbol (^) indicates the centers of rotation of the two-column radials. (**B**) Graph showing the length (mean ± standard deviation) of the rows of radials 1–8 of the medial fin sector and 10–16 in the lateral sector; row 9 is the reference plane of the column turn in the horizontal plane. The dotted vertical line corresponds to the point of the radial sequence at which the two columns within the radial rotate in the horizontal plane.

The stiffness of the segment therefore depends on several factors: 1.the number of radials; 2. the number of columns within the radial (mono-, bi-, or multicolumnar); 3. the variable length of the aligned radials (Figure 6A). This radiograph shows a medial and a lateral sector separated by a transverse line of radials that appear to have a doubling of the columns. This apparent doubling is a projection artifact: the medial sector consists of two parallel columns paired in the dorso-ventral plane. Therefore, the perceived doubling results from the rotation of the two columns in the horizontal plane [22,23]. The mean radial length of the coupled columns of the medial sector is significantly greater than that of the lateral sector (Figure 6B), indicating a higher stiffness in this part of the fin, as the column pairs are firmly connected at the ends by calcified articular discs. Analysis of the radial’s lengths along each medial–lateral line shows a progressive decrease (Figure 6B), further increasing the flexibility of the fin in the lateral sector.

**Figure 7:**
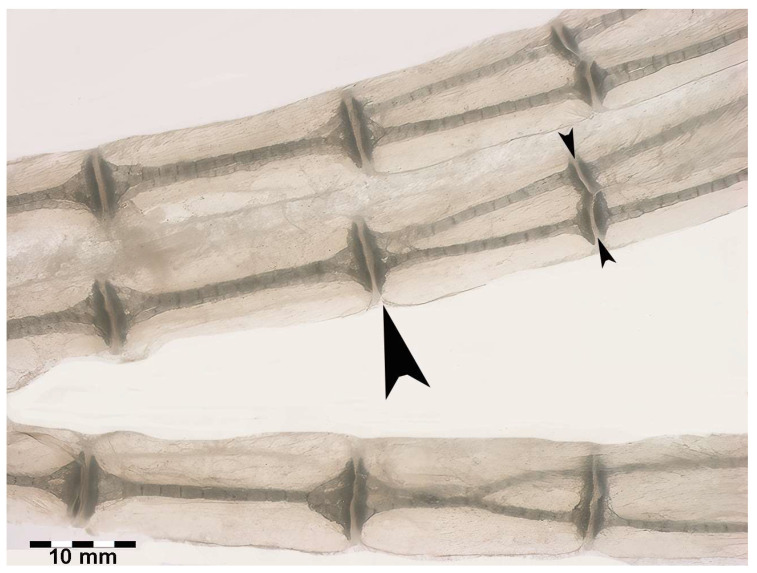
Transillumination and rotational dynamics of dorso-ventral columnar structures in *R. asterias*. Transillumination in dorso-ventral projection of the row plane, in which the dorso-ventral, superimposed columns rotate in the horizontal plane. The paired columns are held in the dorso-ventral position by the medial and lateral single discs (large arrowhead), while the lateral disc cleavage (small arrowheads) allows the columns to rotate within the visco-elastic muff of the non-calcified cartilage.

Transillumination in dorso-ventral projection showed that the rotation of the columns in *R. asterias* is determined by the splitting of two independent joints laterally, while the median joint is single (see Figure 7 below).

**Figure 8:**
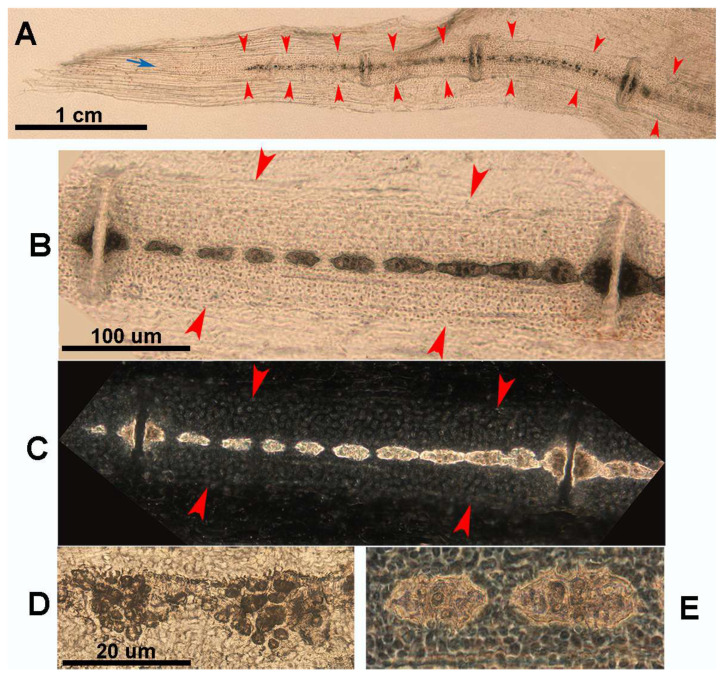
Growth and mineral deposition in *R. asterias* pectoral fin radial, age group C. (**A**) Transilluminated, undecalcified specimen of the apical line of the pectoral fin rays. Mineral deposits in the form of small dark particles surround the aligned tesserae of the central column and the plates at the ends. The profile of the non-calcified cartilage cylinder is marked by red arrowheads, while the distalmost radial is still completely uncalcified. The apical fin consists of a bundle of keratin fibers between the dorsal and ventral tegument (blue arrow). (**B**) Transilluminated, undecalcified specimen of a radial in an advanced stage of calcification. The mineral deposits are compacted and form the aligned tiles of the central column with a tendency to fuse inwards. (**C**) Phase contrast image of B, confirming the pattern of the radial calcification process. (**D**) Transmitted light image, undecalcified specimen of the tiles at higher magnification. The mineral is deposited in the matrix around chondrocytes that are larger than those in the neighboring non-calcified matrix. (**E**) Phase contrast image of (**D**), showing the large chondrocytes embedded in the mineralized matrix.

The observation of juveniles of R. asterias in transmitted light and phase contrast made it possible to document the pattern of mineral deposition in the lateral sector of the fin rays (see Figure 8 below).

**Figure 9:**
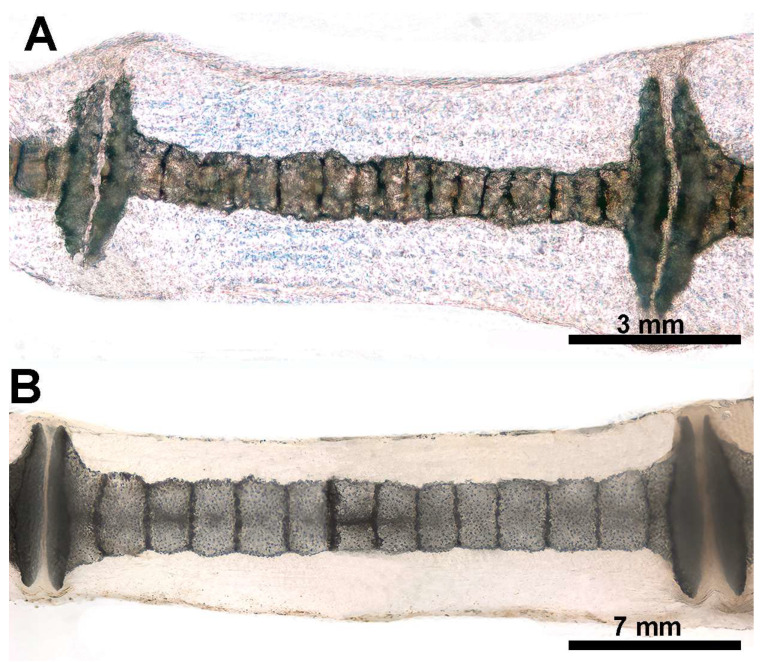
Heat-deproteinated radials of *R. asterias* pectoral fins of age group D (specimens 13 and 15). (**A**) Compacted mineral deposits outline the chained pattern of radial tiles in the center, but their shape is still irregular, as are the platelets at the ends. (**B**) More advanced mineralization with a regular, cylindrical shape of the tiles and plates. Regular distribution of lacunae in the calcified matrix of the plates. The dark bands separating the tiles correspond to carbon deposits of non-calcified cartilage burnt by heat treatment.

The heat deproteination was used to compare the mineralized arrangement of the aligned calcified tiles in the radials of age groups C and D (see Figure 9 below).

### 3.4. Comparison with Marine Mammals

**Figure 10:**
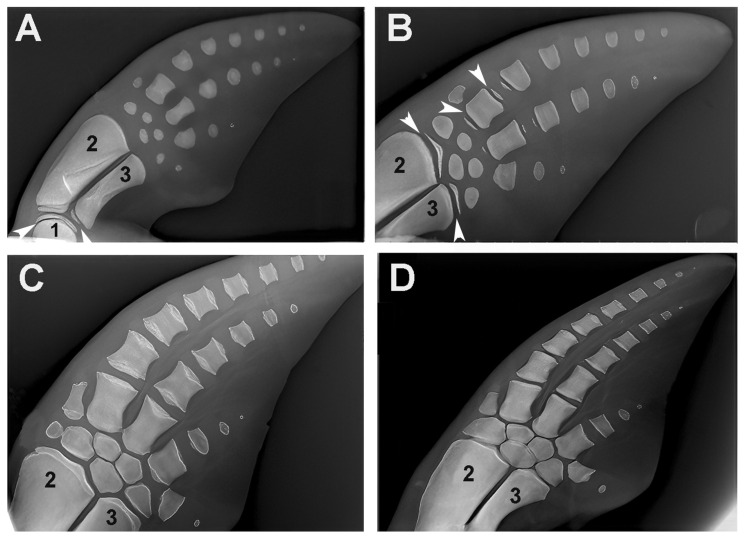
Growth and ossification pattern of dolphin (*Tursipius truncatus*) fins (image reproduced from [7]. (**A**) The stylopodium (1) and the two zeugopodium elements, radius (2) and ulna (3), show the ossified diaphyseal centers with flat epiphyseal ossification centers. The carpal bones, the metacarpals and the distal phalanges each have a single ossification center. White arrows indicate the distal metaphyseal growth plate cartilage of the humerus (1) and the proximal metaphyseal growth plate cartilage of the ulna (3). (**B**) The distal epiphyseal center has also developed in the radius and ulna, the proximal and distal epiphyseal centers in two metacarpals and only the proximal epiphyseal center in the first phalanges. The transverse radio-transparent lines between the diaphyseal and epiphyseal ossification centers correspond to the metaphyseal growth plate cartilage (as in terrestrial mammals) and regulate the longitudinal growth of the skeletal segment. White arrows indicate the distal metaphyseal cartilages of the radius (2) and ulna (3), as well as the proximal and distal cartilages of the 1st metacarpal. (**C**) Early fusion of the diaphyseal and epiphyseal ossification centers due to a halt in chondrocyte proliferation in the clefts of the metaphyseal growth plate. (**D**) Completed growth of the skeleton. The complete fusion of the metaphyseal cartilages indicates arrest of longitudinal growth. Comparison of the lateral and longitudinal diameter of metacarpals and phalanges indicates that the contribution of the metaphyseal plates of marine mammals to the longitudinal growth of the segment is smaller than in terrestrial mammals.

A comparison of the skeletal growth of dolphin flippers with human upper limbs [7] shows the homology with the single proximal segment (stylopodium = humerus), the double medial segments (zeugopodium = radius and ulna) and the distal segments (autopodium = several distal elements: carpal bones, cylindrical metacarpal bones and phalanges) (Figure 10). Although the proximal segments are significantly shortened in dolphins, they retain the skeletal structure typical of mammals, with a diaphyseal ossification center, and two epiphyseal cores at the extremities separated by a radio-transparent line corresponding to the metaphyseal growth plate cartilage. The evolutionary changes in dolphins are characterized by an increased number of distal segments of the autopodium, with no differentiation between metacarpals and phalanges (Figure 10). This pattern bears some resemblance to the high number of aligned elements in the pectoral fins of the Rajidae.

## 4. Discussion

Cartilage plays a key role in the ontogeny of the vertebrate skeleton, as this tissue differentiates earlier than bone in the embryo of both fish and mammals, first modeling the shape of skeletal anlagen and then serving as a precursor to endochondral ossification. The first evidence of embryonic tissues adaptation to mechanical requirements is the notochord, whose cells synthetize and store type 2 collagen matrix, which is thought to be a cartilage-type collagen [2]. This structure is destined to disappear early in vertebrates, while skeletal growth in mammals progresses with a combined pattern of periosteal and endochondral ossification until the calcified cartilage scaffold is gradually replaced by bone until the growth ceases. However, among the jawed vertebrates (Gnathostomata Superclass), the class Chondrichthyes has developed a particular adaptive strategy with a growth pattern that differs from the mammal’s endochondral ossification. The first is characterized by the “tessellated” calcification that does not undergoes remodeling [24,25,26,27].

The structural adaptation to the mechanical requirements of locomotion has stimulated a remarkable and sustained interest in the bony skeleton of mammalians, from the “Wolff’s Law” [28,29,30] to the most recent computational mechano-biological studies [31,32]. Our interest in comparative morphology of skeletal development and growth of mammalians and Batoidea stems from the availability of histological documentation of skeletal growth of *H. sapiens* and the un-remodeled calcified cartilage of Rajidae. Earlier morphology studies of *R. asterias* pectoral fin radials have documented the following layout in the appendicular skeleton in the medial–lateral direction: (a) bi-columnar radials stiffly tied together in the dorso-ventral plane by the two disk plates at the extremities; (b) the two columns’ rotation into the horizontal plane at about half of the fin length (made possible by the lateral disk cleavage and by the visco-elasticity of the un-calcified cartilage muff around the calcified columns; and (c) complete separation of the medially paired columns enabled by the medial disk further splitting combined with the complete separation of the un-calcified cartilage muff; (d) in this way, the lateral fin sector is transformed into a more flexible surface of mono-columnar radials [22]. Therefore, the whole fin works as a mechanical system with a stiff pterygial arc highly movable on the pectoral girdle because of the respective ball–socket and condylar joints, while the large number of aligned radials joined longitudinally by amphiarthroses and connected at the sides by the transversal inter-radial membrane provides variable flexibility along the fin’s whole width. The bi-columnar, medial sector provides a stiffer and stronger support for flapping, while the greater pliability of the mono-columnar, lateral sector and the marginal strip of the fin with an internal structure made only by the keratin fibers between the dorsal and the ventral skin assure significant flexibility for the fin undulating movement.

In both mammalians and chondrichthyans, the early limb and fin cartilage anlagen (prior to the mineral deposition onset) are modeled by the frequency of chondrocyte mitoses, zonal density and mitotic spindle orientation [3,4], a pattern also confirmed by the observed trend of chondrocytes arranging themselves in rows [33,34,35]. The columnar arrangement of chondrocytes is much more evident in the mammalian metaphyseal growth plate cartilages, where the columns are densely packed and oriented along the bone longitudinal axis. However, typical chondrocyte rows are also evident in the *R. asterias* radials anlagen, which grow independently of a previously established calcified scaffold but with the number, density and orientation not so regular as in in the metaphyseal growth plates [22,23]. The fundamental difference in skeletal development and stiffening between mammals and chondrichthyan fishes is that in the former, calcified cartilage is a provisional tissue destined to be reabsorbed and replaced by bone [21,36], whereas in the latter, it forms the definitive skeletal structure and does not undergo remodeling.

The mechano-stat theory explains the skeletal framework as an adaptation to the mechanical requirement of static and dynamic loads generated by locomotion on the ground, in the air or in the water column, but no direct evidence thus far has been provided to demonstrate how mechanical forces can initiate and control the chondrocyte mitotic activity and the 3D mineral deposition layout in the cartilage anlagen. In this context, the Chondrichthyes calcifying cartilage provides a peculiar and interesting model of skeletal segments’ stiffening pattern, allowing skeletal growth and locomotion mechanics to be correlated with the genome, evolution and natural selection [37]. The calcified matrix of the skeleton (cartilage or bone) is characterized not only by the tissue physical properties, such as mineral density, crystalline system, hardness, and flexibility, but also by the physiology and metabolism of the respective vital tissues. Concerning the latter two points, the difference between the bone and calcifying cartilage is that in the first, the osteoblasts survive as osteocytes in the expanding calcifying bone matrix mass (by themselves produced) because both the vascular and canalicular systems develop under a coordinated plan between matrix synthesis and mineral deposition [3,21,38]. In contrast, the chondrocytes embedded in the structurally calcified cartilage are less efficiently nourished by the diffusion of interstitial fluids in a collagen type 2 matrix without a canalicular network and can survive due to a less compact texture of the calcified matrix and a scattered presence of non-calcified cartilage zones.

The report of endochondral ossification of dolphin pectoral fins has been included in this article because it provides further evidence of evolutionary adaptations of the skeleton to swimming in marine mammals, which show the same ossification and growth patterns as *H. sapiens*, but with a higher number of aligned elements in the autopodium that increase the pliability of the apical part of the pectoral fin in water. The comparison with the number of aligned radials of the Batoidea *R. asterias* is even more significant. While dolphins and humans share the common skeletal developmental pattern of endochondral ossification, *R. asterias* represents a different evolutionary solution to the requirements of specific aquatic locomotion. By making these comparisons, we can better understand the different strategies that each species uses to optimize their skeletal structures for the mechanical requirements of their particular environment. Thus, the reference to dolphin fins can serve as a useful link between the ossification processes in *H. sapiens* and cartilage calcification in *R. asterias* and provide insight into how similar and different evolutionary influences can shape skeletal development in different taxa.

In living marine mammals, radiographs of pectoral fins of young and older dolphins have been analyzed to determine skeletal development and chronological age [7,12,14,15,39], providing data on the reproductive status and population demography of local species. However, these studies provide useful morphological data to highlight and compare the endochondral ossification evolutionary changes in relation to marine environment adaptation. Studies on paleontology, phylogenetics and molecular biology have shown that cetaceans share a common heritage with the artiodactyl clades of terrestrial mammals [40]. Without entering into the evolutionary history of cetaceans, this study develops a comparison of upper appendicular skeletal morphology, growth/development and stiffening patterns between the terrestrial mammalian *H. sapiens*, the chondrichthyan *R. asterias* and the marine mammal *T. truncates* (referring for the latter to the radiographic images of the flipper skeletal segments recently published by [7]). Moreover, the same histomorphology we observed in human cartilage anlagen has been described in the embryonic development of the dolphins’ flipper bones [14], whose subsequent ossification pattern is disclosed by radiographic film images consistent with that of *H. sapiens*, but with differences that support the hypothesis of the structural adaptation to the mechanical demand.

The general developmental scheme of the mammalian skeleton with a single bone element in the stylopodium (humerus), two elements in the zeugopodium (radius and ulna), and several elements in the autopodium (carpals, metacarpals and phalanges) is the same as in *H. sapiens*, as is the distribution of the ossification centers with one diaphyseal and two epiphyseal on the extremities separated by a transverse radio-transparent line (the metaphyseal growth plate cartilage). This makes it possible to distinguish the still-growing elements from those with fused epiphyseal centers (longitudinal growth completed) and also from those at an earlier stage of development, where epiphyseal ossification had not yet begun or was highlighted by limited and shallow calcification. The adaptation of the fin bones to the mechanical requirements of locomotion in the water is documented in today’s dolphin species not only by the reduction in the size of the rays of the autopod digits and the increase in the number of aligned phalanges in the middle rays [13,14] but also by a very early cessation of chondrocyte proliferation in the cartilages of the metaphyseal growth plate of the humerus, radius and ulnas, as indicated by the short length of the stylopodium and zeugopodium elements compared to *H. sapiens* and other land mammals. These two macroscopic features support the theory of mechanical demand adaptation in the water column. The latter is further strengthened by the comparison with the structure of the pectoral fin of *R. asterias* on the other side of the evolutionary scale with the extreme segmentation of the radials, surface enlargement and modulated flexibility as well as the reduction in stylopodium and zeugopodium to the pterygial arch.

The development and evolution of the appendicular skeletal elements takes place at different hierarchical levels of biological organization, the first being in the embryo with cartilage differentiation from the mesenchyme and the growth of the anlagen under the genetic control of chondrocyte proliferation, which is the same in both mammalian and Chondrichthyes skeletons. The stiffening of the cartilage is the subsequent step in the adaptation of the tissue to mechanical stress, which is characterized by the deposition of minerals in the organic matrix of the embryonic anlagen of mammals and Chondrichthyes. The difference between the two classes is that in the former, the calcified cartilage is resorbed and replaced by a bone matrix produced by osteoblasts (endochondral ossification), while in the latter, the embryonic tissue remains and forms the final skeletal structure of the fins, whose growth pattern is documented by the “tesseral” morphology of the fin rays of *R. asterias*.

## 5. Conclusions

The study highlights the key differences in skeletal development between mammals and chondrichthyan fishes. In mammals, cartilage serves as a temporary scaffold that is gradually replaced by bone through endochondral ossification, while in chondrichthyan fishes, such as *R. asterias*, the calcified cartilage remains as the final skeletal structure without remodeling. This distinction reflects different evolutionary strategies to adapt to mechanical requirements, with mammals showing a more dynamic ossification process and chondrichthyans developing a stable, non-remodeled cartilage structure. Comparative studies, such as those on dolphin fins, illustrate how skeletal adaptations to specific environments have evolved differently across taxa.

## Figures and Tables

**Table 1:** Data of the analyzed *R. asterias* juvenile specimens.

No.	Group	Sex	Weight (g)	Pectoral Fins Width (cm) (*)	Total Length (cm)
1	A	n.a.	1.20	4.0	7.6
2	B	n.a.	2.10	5.0	8.2
3	B	n.a.	2.20	5.5	9.2
4	B	n.a.	2.80	6.0	9.6
5	B	n.a.	2.95	6.3	9.7
6	B	n.a.	3.10	6.3	9.9
7	B	n.a.	3.25	6.5	n.a.
8	B	n.a.	4.30	7.6	10.8
9	C	F	8.50	9.9	12.7
10	C	M	9.70	10.8	14.6
11	C	F	9.90	11.7	16.3
12	C	F	10.50	12.5	16.9
13	D	M	260.0	19.0	25.0
14	D	M	350.0	27.5	40.0
15	D	M	769.0	34.0	52.5

n.a. = not assessable. (*) The width of the pectoral fin was measured from the tip of the left pectoral fin to that of the corresponding right fin.

## Data Availability

All the data are present in the article.
